# Retraction Notice

**Published:** 2011

The following article is being retracted as a part of the manuscript was plagiarized.

Moazzam MS, Ahmed SM, Bano S. Traumatic haemorrhage of occult phaeochromocytoma in a patient with septic shock. J Emerg Trauma Shock 2010;3:300.

Editor, JETS

